# Case report: Granulomatosis with polyangiitis patient presented with a mass in the aortic root

**DOI:** 10.3389/fimmu.2024.1373769

**Published:** 2024-11-28

**Authors:** Yinyan Teng, Mei Chen, Zhongwei Chen, Xiaowei Zhang, Zhongyun Li, Shusheng Liao

**Affiliations:** ^1^ Department of Ultrasound, The First Affiliated Hospital of Wenzhou Medical University, Wenzhou, Zhejiang, China; ^2^ Department of Ultrasonography, The First Affiliated Hospital of Ningbo University, Ningbo, Zhejiang, China; ^3^ Department of Radiology, The First Affiliated Hospital of Wenzhou Medical University, Wenzhou, Zhejiang, China

**Keywords:** granulomatosis with polyangiitis, diagnosis, heart, echocardiography, therapy

## Abstract

Granulomatosis with polyangiitis (GPA) is an autoimmune inflammatory disease that affects small- and medium-sized blood vessels in the body, representing a rare entity. Cardiac involvement was identified as an independent risk factor for death in GPA patients, yet it has not been systematically elucidated in previous literature. Cardiac lesions in patients with GPA can manifest in various ways, such as pericarditis, myocarditis, coronary vasculitis, valvular abnormalities, conduction system abnormalities, and heart failure. Herein, we report a 55-year-old woman with GPA; she had a 2-year history of recurrent episodes of headache, accompanying sickness, and fatigue, which have been aggravated for the past half-month. The main manifestation is presenting as a mass in the aortic root, which was successfully diagnosed by multimodality imaging (including two-dimensional echocardiography, contrast-enhanced ultrasound, and computed tomography). After treatment with methylprednisolone and cyclophosphamide, the patient’s symptoms significantly improved and she remained asymptomatic over 6 months of follow-up. This article will enrich our knowledge about cardiac involvement in GPA patients and highlights the value of imaging, especially ultrasound, in the diagnosis and post-treatment follow-up of this condition.

## Introduction

Antineutrophil cytoplasmic antibody (ANCA)-associated vasculitis (AAV) is a kind of systemic necrotizing vasculitis. The main pathological features of AAV are fibrous necrosis and inflammation in the walls of neutrophil and small vessels. Clinical types include granulomatosis with polyangiitis (GPA), eosinophilic granulomatosis with polyangiitis (EGPA), and microscopic polyangiitis (MPA) (1). Symptoms of AAV patients are non-specific, such as arthralgia, weight loss, malaise, and fatigue, approximately 30%–45% of whom manifested as fever (2). Cardiovascular involvement is identified as an independent risk factor for death in AAV patients. According to the study by McGeoch et al. (3), 3.3% of patients with GPA show cardiovascular involvement. Cardiac lesions in patients with GPA are characterized by pericarditis, myocarditis, coronary vasculitis, valvular abnormalities, myocardial infarction, conduction system abnormalities, and heart failure (4–6). Presenting as a cardiac mass is very rare in this disease, but it can be easily misdiagnosed as other diseases such as infection and a malignant tumor, leading to inappropriate treatment. We reported a case of GPA presenting as a mass in the aortic root that was successfully diagnosed by multimodality imaging and timely treated. It will enrich our knowledge of this disease, reduce clinical misdiagnosis, and improve the efficiency of clinical treatment.

## Case presentation

A 55-year-old woman was admitted to our hospital with a 2-year history of recurrent episodes of headache and accompanying dizziness, sickness, and fatigue, which have been aggravated for the past half-month. She also had a history of sinusitis and otitis media for more than 2 years. She denied the history of arthritis and there were no cardiovascular risk factors, such as system hypertension, coronary artery disease, and diabetes mellitus. This time, her clinical symptoms were accompanied by a fever with temperature of 38.7°C and chest distress. Laboratory tests showed dramatic elevated inflammatory blood markers, with an erythrocyte sedimentation rate (ESR) of 160 mm/h (normal range <20 mm/h) and C-reactive protein (CRP) 157 mg/L (normal range <6 mg/L). WBC (5.20×10^9^/L) and platelet (285×10^9^/L) counts were within the normal range. The level of NT-proBNP was higher at 1,069 ng/L (normal range <125 ng/L) and hs-TNT was within the normal range. The level of creatinine was at 49 μmol/L and within the normal range. Diagnostic workup revealed positive pANCA, and the anti-myeloperoxidase (MPO) antibody was increased at 5.2 AI (normal range within 0–0.99 AI). Testing for anti-nuclear antibodies (ANA) was positive with a titer of 1:320; however, anti-double-stranded DNA (dsDNA), anti-SSA, anti-SSB, anti-Scl-70, anti-Jo-1, and anti-Ro52 antibody yielded negative results. The phospholipid antibodies and lupus anticoagulants also showed negative results. Serum IgG4 was at 1.05 g/L, within the normal range. The level of D-dimer was slightly higher at 1.72 mg/L (normal range <0.05 mg/L). Further tests showed positive anti-protein C antibody and 4G/5G plasminogen activator inhibitor-1 (PAI-1) gene. In addition, the results of lymphocyte differential antigen testing analysis reveal a normal ratio of CD4+/CD8+ (of T lymphocytes) at 1.0.

**Figure 1:**
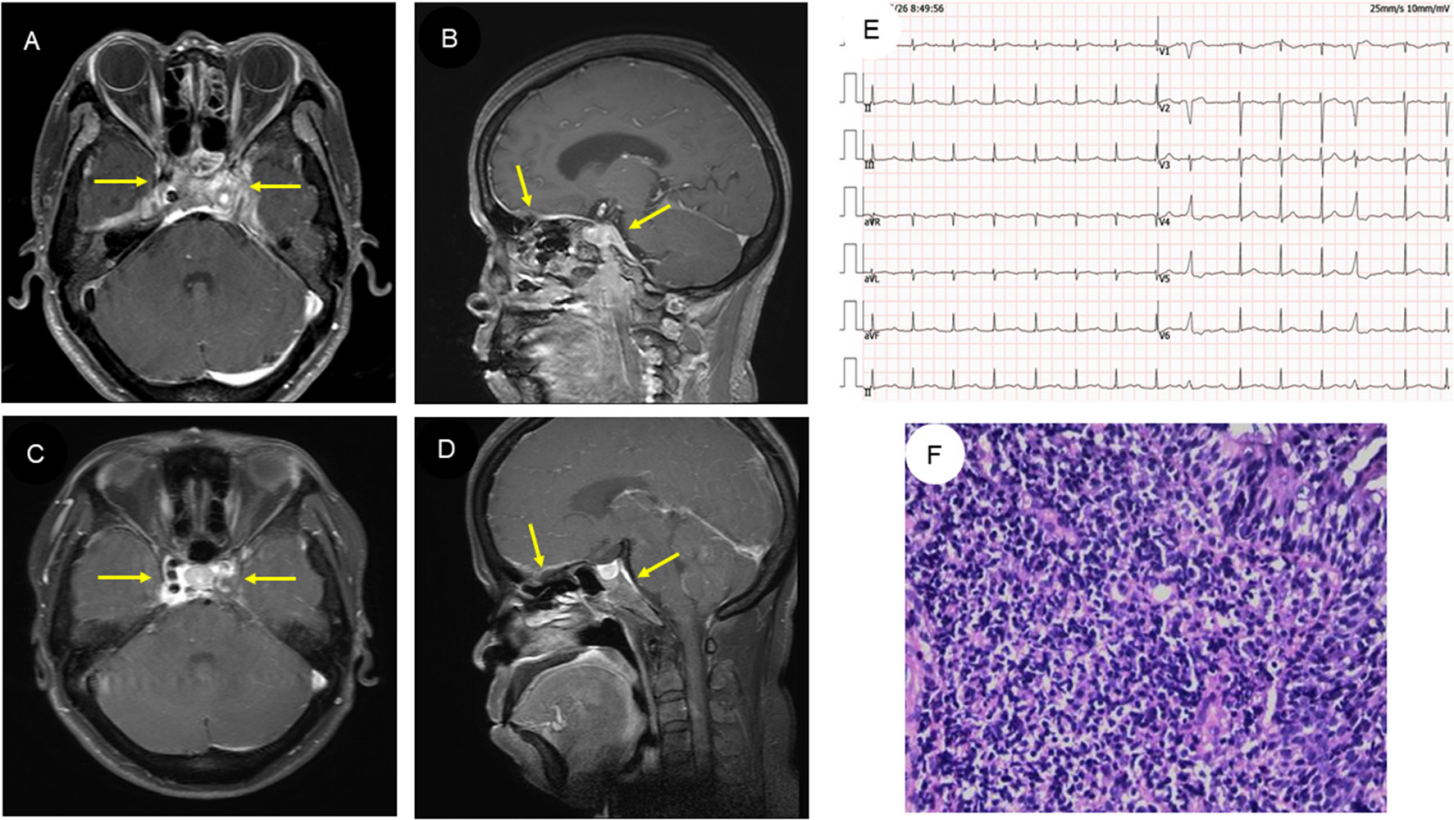
MRI findings, electrocardiogram, and histopathologic results of the patient. **(A, B)** Head MRI of the patient revealed abnormal signals in both the anterior and middle cranial fossa and nasopharynx wall. **(C, D)** Subsequent head MRI showed that the abnormal signal had reduced in size after treatment. **(E)** The electrocardiogram revealed first-degree atrioventricular block. **(F)** Histopathologic examination of the nasopharynx suggests chronic inflammation of the mucosa with reactive lymphoid hyperplasia (HE staining, original magnification ×200).

The electrocardiogram showed a first-degree conduction block (**Figure 1E**). Her head MRI revealed meninges and cavernous sinus thickening in both anterior and middle cranial fossa with abnormal signal, and diffuse signal abnormality in both nasopharynx walls, parapharyngeal space, bilateral long cephalic muscle, medial pterygoid muscle, and lateral pterygoid muscle (**Figures 1A, B**). Nasopharyngeal tissue biopsy was performed, and histopathology showed consistency with chronic inflammation of the mucosa with reactive hyperplasia of lymphoid tissue (**Figure 1F**). Her chest computed tomography (CT) scan revealed an irregular low-density mass in the aortic root (**Supplementary Figures S1A, B**); upon enhancement scanning, the lesion displayed low enhancement (**Supplementary Figures S1D, E**). In addition, a few pericardial effusion and bilateral pleural effusion were found.

**Figure 2:**
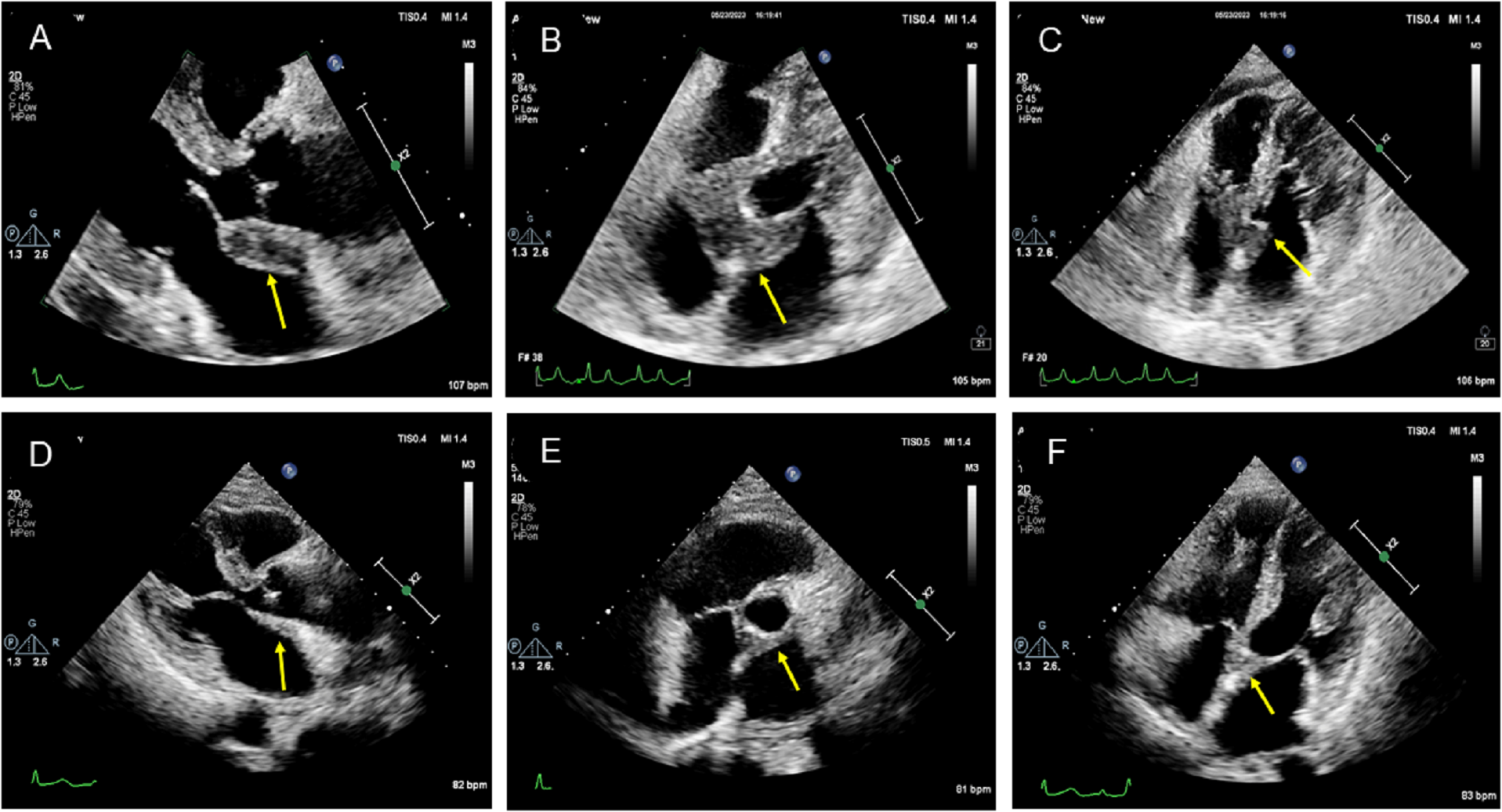
Two-dimensional transthoracic echocardiographic manifestation of the lesion in the aortic root. **(A–C)** Two-dimensional transthoracic echocardiography revealed an isoechoic mass around the aortic root with a clear boundary, irregular shape, uneven internal echo, and no calcification. **(D–F)** Transthoracic echocardiography showed that the mass around the aortic root was smaller after treatment.

**Figure 3:**
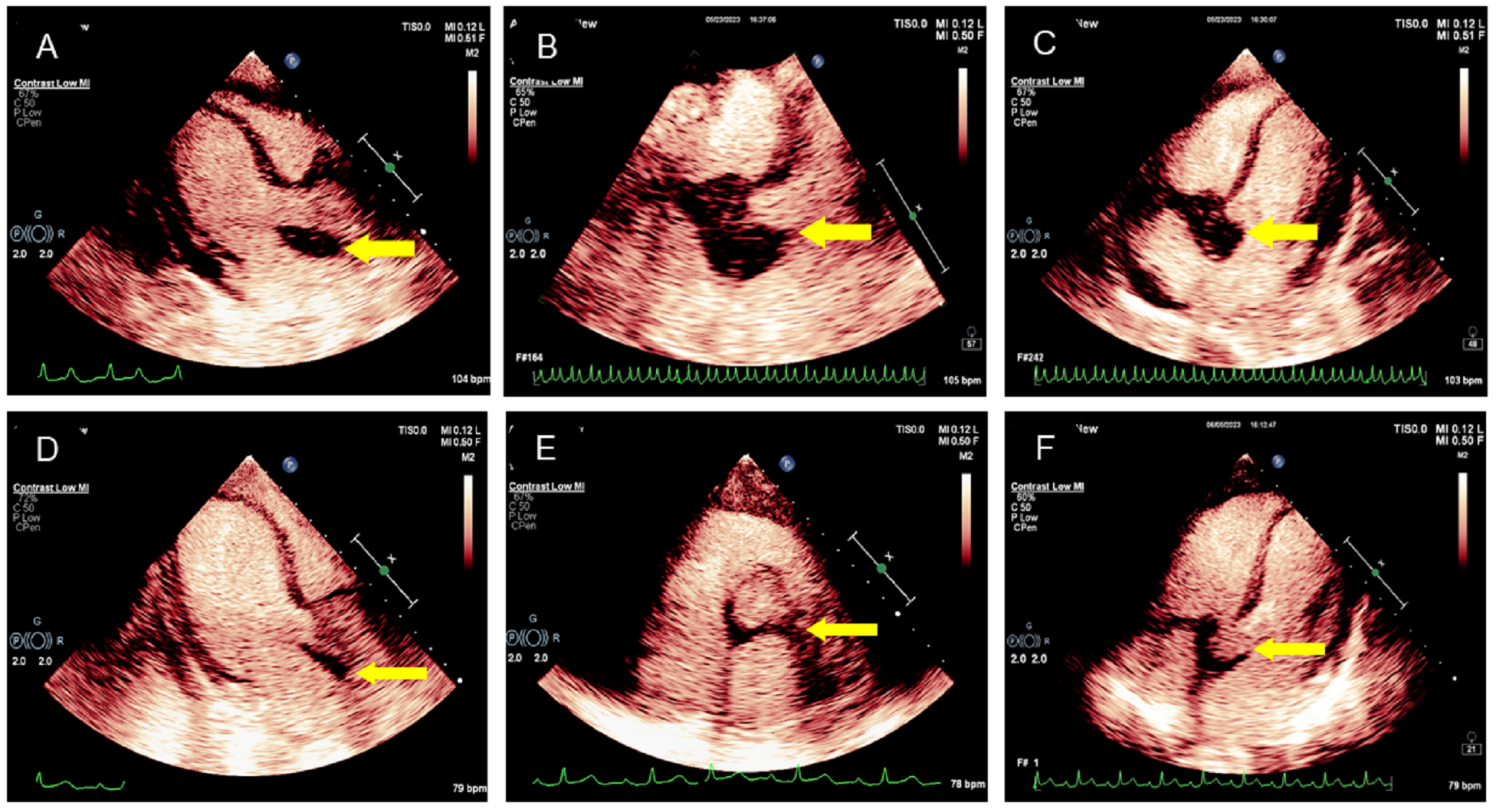
CEUS manifestation of the lesion in the aortic root. **(A–C)** CEUS demonstrated the mass surrounding the aortic root with a low enhancement pattern; the degree of enhancement was lower than that of the surrounding myocardium. No necrotic area was found in the mass. **(D–F)** CEUS showed that the lesion in the aortic root was smaller after treatment.

The patient underwent transthoracic echocardiography and presented with an iso-echoic mass of approximately 38 × 42 × 27 mm around the aortic root, with a clear boundary, irregular shape, and uneven internal echo without calcification (**Figures 2A–C**, **Supplementary Movies 1–3**). Color Doppler demonstrated mild-to-moderate aortic regurgitation. In addition, a small of amount of pericardial effusion was found. However, her cardiac function was preserved with a normal LVEF at 66%. To further understand the perfusion characteristics of the lesions, the patient underwent contrast-enhanced ultrasound (CEUS). The CEUS demonstrated that the mass showed a low enhancement pattern, with less enhancement than the surrounding myocardium, and washed out earlier than the myocardium. There was no necrotic area in the mass (**Figures 3A–C**, **Supplementary Movies 4–6**). Scanning of the carotid artery revealed thrombosis in the left internal carotid artery (**Supplementary Figures S2A–D**). In addition, the examination of bilateral superficial temporal artery (STA) was also carried out, and no abnormalities were found on ultrasound (**Supplementary Figures S2E–J**).

**Figure 4:**
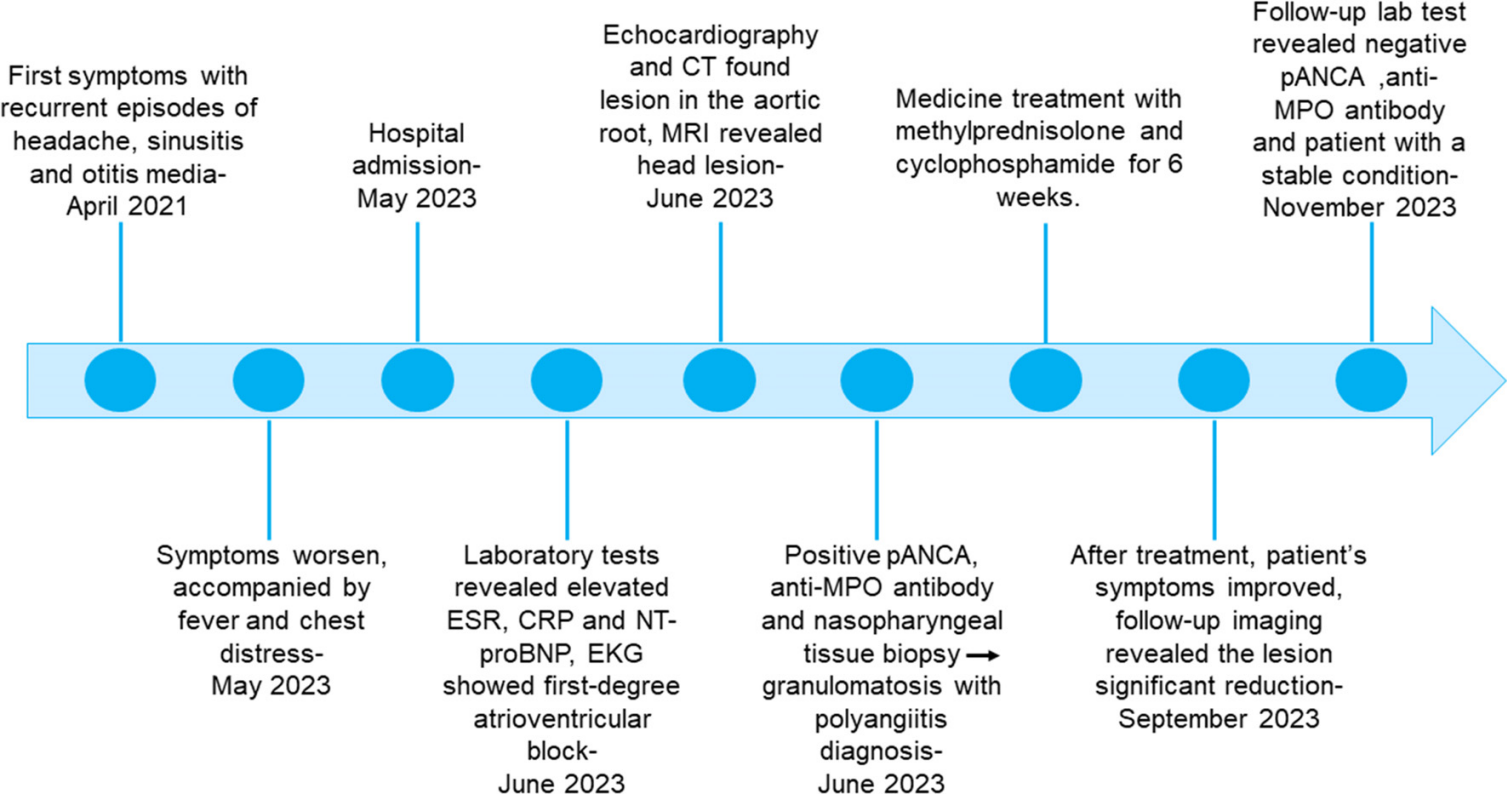
Timeline.

The initial diagnosis considered for the patient was ANCA-associated vasculitis and GPA. Meanwhile, she was also considered to have thrombophilia. During hospitalization, therapy with methylprednisolone was started at 1,000 mg daily, resulting in a good clinical and biochemical response. Subsequently, the methylprednisolone was gradually reduced to 40 mg daily. Following this corticoid tapering schedule, cyclophosphamide was administered at 600 mg for immune suppression. In addition, considering that this patient had thrombophilia, heparin (0.4 mL) was used daily as anticoagulation therapy for 1 week, which was subsequently changed to rivaroxaban at 10 mg daily. Antiplatelet therapy with clopidogrel (75 mg daily) was also arranged for her. After 6 weeks of medicine treatment, the patient’s hearing improved, although tinnitus persisted and headache significantly improved. A follow-up MRI showed a significant reduction in the lesion (**Figures 1C, D**). Echocardiographic follow-up revealed a significant reduction in the mass in the aortic root compared to the initial presentation (**Figures 2D–F**, **3D–F**, **Supplementary Movies 7–12**). After 3 months of therapy, a chest CT scan demonstrated the disappearance of the lesion in the aortic root (**Supplementary Figures S1C, F**). Carotid artery ultrasound examination showed regression of the thrombosis in the left internal carotid artery. The patient was followed up for 6 months as outpatient, and laboratory tests showed negative pANCA and anti-MPO antibody, and normal levels of ESR (11 mm/h), CRP (2.2 mg/L), and NT-proBNP (164 ng/L), indicating a stable condition (**Figure 4**).

## Discussion

GPA (formerly known as Wegner’s granulomatosis) is an autoimmune inflammatory disease that affects small- and medium-sized blood vessels in the body. The disease is uncommon, with a reported incidence of 5–10 cases per million population (7). It primarily affects people aged 40–60 years; our case falls within this age range. GPA can affect every organ in the body, primarily involving the respiratory tract, lung, and kidney, while cardiac involvement is relatively rare and mostly subclinical. According to previous literature reports, cardiac involvement with clinical symptoms was observed in 3.3%–10.3% of patients with GPA (8, 9). At present, the mechanism of GPA-induced cardiac damage has not been accurately reported, but it may be related to the acceleration of arteriosclerosis, vascular inflammatory response, and related complications. Studies have suggested that myocardial injury in GPA patients may be caused by vasculitis, leading to coronary arteritis and occlusion of coronary arteries. Alternatively, it may be caused by eosinophil granulocyte infiltration in the myocardium and endocardium, and activation and release of cytotoxic granular protein and lipid mediators, which are directly caused by myocardial injury (10). Cardiac lesions in patients with GPA are commonly characterized by valvular abnormalities, pericarditis, myocarditis, coronary vasculitis, heart conduction system abnormalities, and intracardiac mass. The most common cardiac manifestations in GPA patients include pericarditis, aortic regurgitation, and conduction system abnormalities (3). In this case, a first-degree atrioventricular block was observed. Complete atrioventricular block can also be found in some conditions (11–13). In recent years, literature reports indicate that cardiac valvular involvement in GPA has become more common. Among these cases, the aortic valve is most commonly affected, followed by the mitral valve and combined aortic and mitral valve disease. Valve regurgitation is the most common valvular lesion. Our case also presented mild-to-moderate aortic regurgitation. Histopathologic examination of valvular lesion in GPA patients shows fibrinoid connective tissue damage and fragmentation of the collagen fibrils, suggesting ANCA-induced endothelial injury through the activation of neutrophils (14). If the GPA patient presented cardiovascular signs and symptoms, the cardiac involvement should be suspected (**Supplementary Figure S3**).

GPA is a complex and potentially fatal disease with high mortality if not treated promptly. Early detection and prompt treatment can improve prognosis and reduce mortality (15). Conventional ultrasound, as a noninvasive means of imaging examination, has the advantages of being economical and simple and causing no radiographic damage, making it easily accessible. It is widely used in clinical practice and is often the initial examination method for the evaluation of cardiac involvements in this disease. Two-dimensional echocardiography can identify structural abnormalities of the heart and pericardial effusion, while Doppler echocardiography can assess the cardiac valvular function.

As a new imaging technology, CEUS is simple, reliable, and safe. It enables dynamic observation of the microcirculation inside the mass in real time and has unique value in the diagnosis and differential diagnosis of the lesion. The presence of an aortic root space-occupying lesion in this patient is an extremely rare manifestation of cardiac involvement in GPA patients. Only a few reports in the literature have described such findings. Two middle-age patients with GPA presented with a mass involving the aortic outflow tract or intracardiac septum and mitral valve (16, 17), and two pediatric patients presented with an intracardiac mass in the left ventricle (18, 19). To the best of our knowledge, no CEUS findings of GPA have been reported in the previous literature, and our patient showed that the performance of GPA on CEUS has certain characteristics. This article provided strong evidence of cardiac involvement through two-dimensional echocardiography and CEUS, offering definitive imaging evidence for the follow-up of patients after treatment. Moreover, the chest CT scan of our patient further corroborated the imaging information regarding cardiac involvement. Generally, corticosteroid therapy remains the first drug of choice in GPA, and immunosuppressants should be added in patients with cardiac involvement. For our case, two therapeutic approaches were simultaneously employed, leading to a satisfactory outcome during a 6-month follow-up. After treatment, symptoms of the patient were alleviated, and both the cardiac lesion and thrombosis in the left internal carotid artery showed a significant improvement. In addition, immunobiological therapy (such as rituximab) may be considered for GPA patients with conduction system abnormalities.

## Conclusion

GPA is a rare disease, and it can affect virtually any organ. The heterogeneous manifestations of GPA with cardiac involvement represent a significant challenge in the diagnosis of this condition. The present case showed that cardiac imaging, especially echocardiography, is useful for the diagnosis of cardiac impairment caused by GPA. This case report enriched our understanding of the imaging findings of cardiac involvement in patients with GPA. Cardiac involvement in GPA often indicates that the disease is active. However, if diagnosed promptly and accurately, and treated actively with corticosteroids and immunosuppressants, it can effectively alleviate symptoms and improve the prognosis of patients.

## Supplementary material

Supplementary Figure S1 Chest CT manifestation of the lesion in the aortic root. **(A, B)** Chest CT scan revealed a low density lesion in the aortic root. **(D, E)** CT enhanced scan demonstrated the lesion with low enhancement. **(C, F)** Chest CT scan showed that the lesion was not found after treatment.

Supplementary Figure S2 Ultrasound examination of left internal carotid artery and bilateral superficial temporal artery (STA). **(A, B)** Two-dimensional ultrasound revealed thrombosis in the left internal carotid artery. **(C)** CDFI showed changes in blood flow within the carotid artery lumen. **(D)** CEUS indicated the thrombosis in internal left carotid artery showed no contrast agent perfusion. **(E, F)** CDFI demonstrated normal blood flow within the right superficial temporal artery. **(G)** Pulse-wave(PW) spectral Doppler interrogation revealed the normal velocity of right superficial temporal artery. **(H, I)** CDFI demonstrated normal blood flow within the left superficial temporal artery. **(J)** PW interrogation revealed the normal velocity of left superficial temporal artery.

Supplementary Figure S3 The diagnostic algorithm for patients with susceptive GPA (of whom cardiac involvement).
