# Supplementary figures and images for: Case report: Granulomatosis with polyangiitis patient presented with a mass in the aortic root
Source: Front Immunol. 2024 Nov 28;15:1373769. doi: 10.3389/fimmu.2024.1373769 (PMC11635199; doi:10.3389/fimmu.2024.1373769)

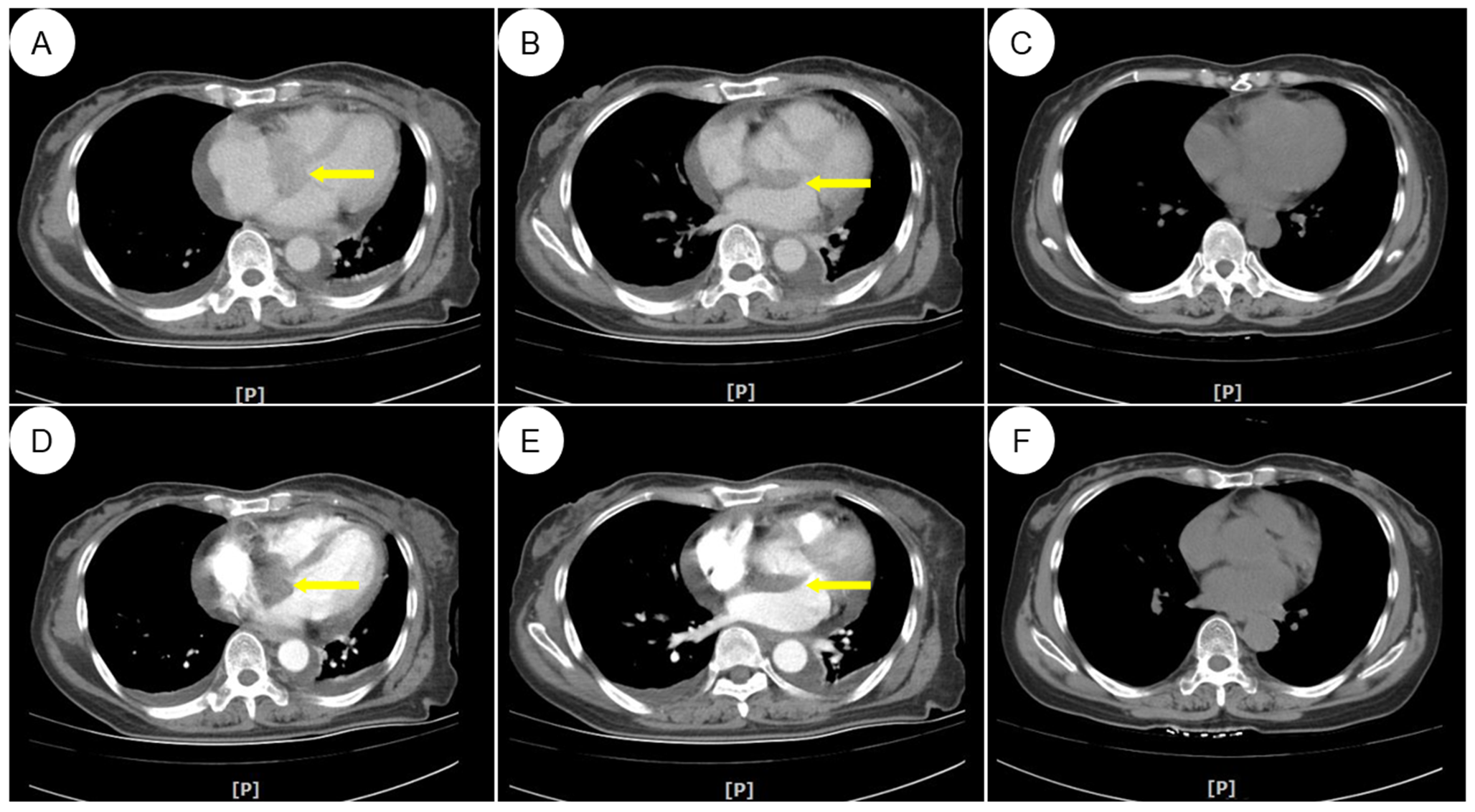

Supplement: Supplementary Figure S1 — Chest CT manifestation of the lesion in the aortic root. (A, B) Chest CT scan revealed a low density lesion in the aortic root. (D, E) CT enhanced scan demonstrated the lesion with low enhancement. (C, F) Chest CT scan showed that the lesion was not found after treatment. [file Image1.tif]

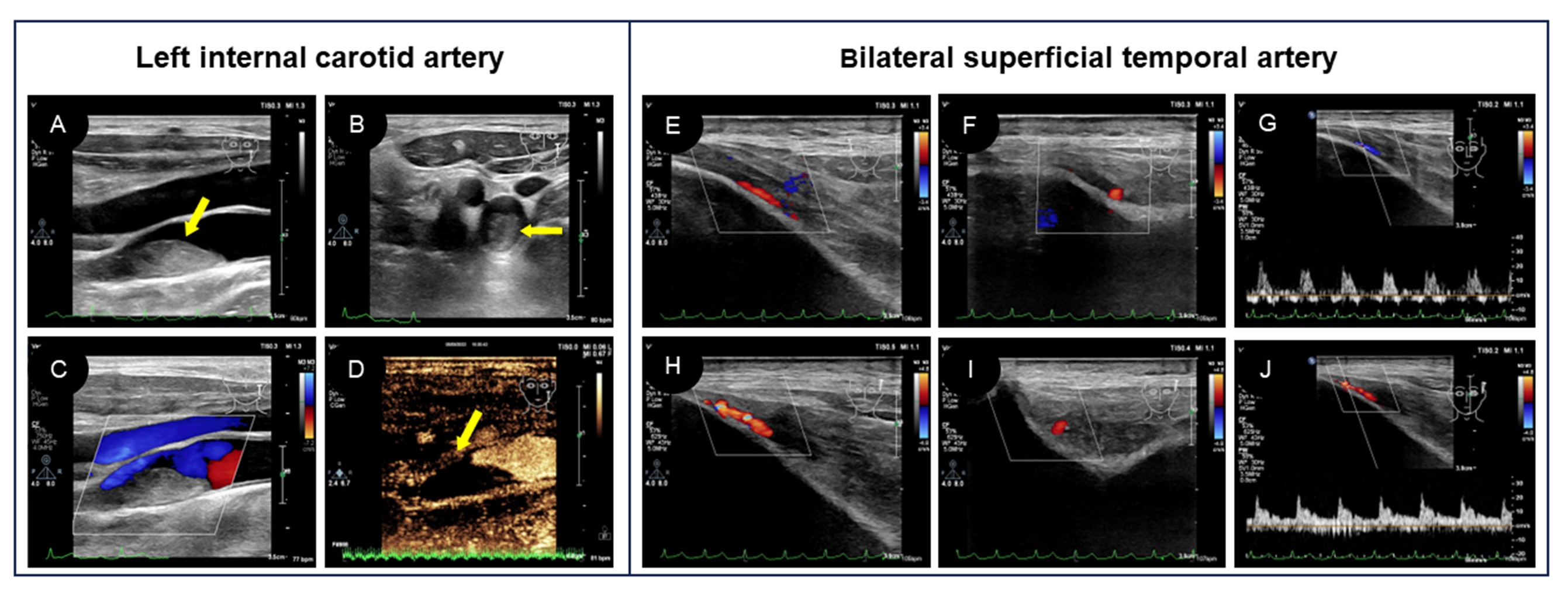

Supplement: Supplementary Figure S2 — Ultrasound examination of left internal carotid artery and bilateral superficial temporal artery (STA). (A, B) Two-dimensional ultrasound revealed thrombosis in the left internal carotid artery. (C) CDFI showed changes in blood flow within the carotid artery lumen. (D) CEUS indicated the thrombosis in internal left carotid artery showed no contrast agent perfusion. (E, F) CDFI demonstrated normal blood flow within the right superficial temporal artery. (G) Pulse-wave(PW) spectral Doppler interrogation revealed the normal velocity of right superficial temporal artery. (H, I) CDFI demonstrated normal blood flow within the left superficial temporal artery. (J) PW interrogation revealed the normal velocity of left superficial temporal artery. [file Image2.tif]

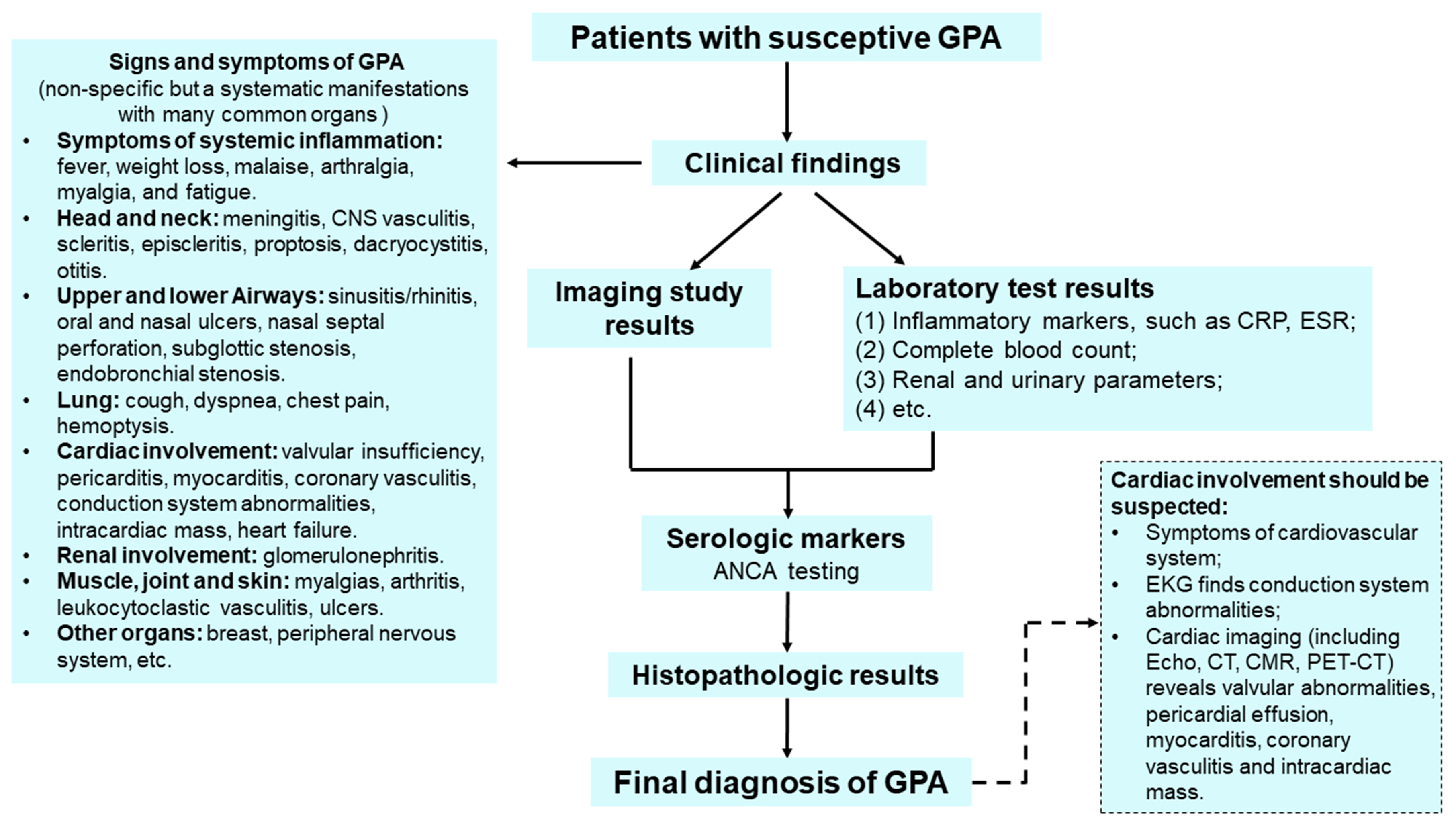

Supplement: Supplementary Figure S3 — The diagnostic algorithm for patients with susceptive GPA (of whom cardiac involvement). [file Image3.tif]
